# Considering LTSS Through the Lenses of Social Construction of Target Populations: Theory and Neoliberalism

**DOI:** 10.1093/geroni/igaa057.115

**Published:** 2020-12-16

**Authors:** Tommy Buckley, Carole Cox, Israel (Issi) Doron

**Affiliations:** 1 Virginia Commonwealth University, Richmond, Virginia, United States; 2 Fordham University, New York, New York, United States; 3 University of Haifa, Haifa, Israel

## Abstract

The global increase of the older population has led to a greater demand for long term support services (LTSS) that address their rights and needs for care. However, policies among countries remain diverse with varying options, services, and recognition of human rights. This study applies the Social Construction of Target Population (SCTP) theory which relates to the perception of older adults and Neoliberalism, a political theory associated with policies of economic privatization, deregulation, and free market activity to the analysis of LTSS systems. As an example, in the United States, the concept of Successful Aging, conflicts with the need for LTSS while present Neoliberal policies that stress minimal government intervention and regulation contribute to little support for any comprehensive LTSS policy. This paper reviews existing policies in 7 countries, representing diverse regions, cultures, and political systems as a means of understanding the relationship of SCTP and Neoliberalism to LTSS. Highlighting the roles that these theories may play can assist in understanding and developing systems that meet the rights and health care needs of older adults.

